# Impact of Pre-existing Anti-polyethylene Glycol Antibodies on the Pharmacokinetics and Efficacy of a COVID-19 mRNA Vaccine (Comirnaty) In Vivo

**DOI:** 10.34133/bmr.0112

**Published:** 2024-12-11

**Authors:** Yen-Ling Liu, Tzu-Yi Liao, Kai-Wen Ho, En-Shuo Liu, Bo-Cheng Huang, Shih-Ting Hong, Yuan-Chin Hsieh, Mu-Shen Chang, Bing-Tsung Wu, Fang-Ming Chen, Steve R. Roffler, Chiao-Yun Chen, Yuan-Chieh Yang, Tian-Lu Cheng

**Affiliations:** ^1^ Graduate Institute of Medicine, College of Medicine, Kaohsiung Medical University, Kaohsiung, Taiwan.; ^2^ Drug Development and Value Creation Research Center, Kaohsiung Medical University, Kaohsiung, Taiwan.; ^3^ School of Medicine for International Students, I-Shou University, Kaohsiung, Taiwan.; ^4^ PhD Program in Life Science, College of Life Science, Kaohsiung Medical University, Kaohsiung, Taiwan.; ^5^ Department of Radiation Oncology, Faculty of Medicine, College of Medicine, Kaohsiung Medical University, Kaohsiung, Taiwan.; ^6^ Institute of Biomedical Sciences, Academia Sinica, Taipei, Taiwan.; ^7^ Department of Medical Imaging, Kaohsiung Medical University Hospital, Kaohsiung, Taiwan.; ^8^ Department of Laboratory Medicine, Kaohsiung Municipal United Hospital, Kaohsiung, Taiwan.; ^9^ Department of Biomedical Science and Environmental Biology, Kaohsiung Medical University, Kaohsiung, Taiwan.

## Abstract

The presence of anti-polyethylene glycol (anti-PEG) antibodies can hinder the therapeutic efficacy of PEGylated drugs. With the widespread use of a PEGylated coronavirus disease 2019 (COVID-19) messenger RNA vaccine (Comirnaty), the impact of pre-existing anti-PEG antibodies on vaccine potency has become a point of debate. To investigate this, we established mouse models with pre-existing anti-PEG antibodies and divided them into 3 groups: group 1 with anti-PEG immunoglobulin G + immunoglobulin M concentrations of 0.76 to 27.41 μg/ml, group 2 with concentrations of 31.27 to 99.52 μg/ml, and a naïve group with no detectable anti-PEG antibodies. Results indicated that anti-spike antibody concentrations significantly decreased in group 1 and group 2 after the 2nd vaccine dose compared to those in the naïve group. Spearman’s rank correlation analysis demonstrated a negative relationship between anti-spike antibody production and anti-PEG antibody levels at both the 2nd and 3rd doses (2nd dose: *ρ* = −0.5296, *P* = 0.0031; 3rd dose: *ρ* = −0.387, *P* = 0.0381). Additionally, spike protein concentrations were 31.4-fold and 46.6-fold lower in group 1 and group 2, respectively, compared to those in the naïve group at 8 h postvaccination. The concentration of complement C3a in group 2 was significantly higher than that in the naïve group after the 3rd dose. These findings confirm that pre-existing anti-PEG antibodies diminish vaccine efficacy, alter pharmacokinetics, and elevate complement activation. Therefore, detecting pre-existing anti-PEG antibodies is crucial for optimizing vaccine efficacy, ensuring patient safety, and developing improved therapeutic strategies.

## Introduction

Polyethylene glycol (PEG) is a versatile polymer that is commonly incorporated into PEGylated nanoparticle drugs to increase their biocapabilities [1]. The Moderna mRNA-1273 (Spikevax) [2] and Pfizer–BioNTech (Comirnaty) [3] coronavirus disease 2019 (COVID-19) messenger ribonucleic acid (mRNA) vaccines have demonstrated beneficial efficacy for preventing an uncontrolled outbreak of COVID-19 [4]. Because mRNA is liable to be degraded by ribonucleases in the circulation, mRNA vaccines conventionally encapsulate mRNA into lipid nanoparticles (LNPs) as a delivery vehicle [5,6]. The LNPs contain PEG-lipids that help LNPs disperse in aqueous solutions and prevent aggregation during storage [7]. PEG has been considered a hydrophilic, dynamic, nontoxic, and low-immunogenicity polymer. Due to these properties, PEG molecules received approval from the US Food and Drug Administration (FDA) in the early 1970s [8]. PEG is widely used in daily necessities such as cosmetic products, lubricant eye drops, and moisturizing lotions [9]. In the 1990s, PEGylation of biopharmaceuticals was also approved by the FDA. PEGylation is the process of attaching PEG to molecules, mainly peptides [10], proteins [11], and antibody fragments [12], which can enhance the safety and stability of many therapeutics. In the clinic, PEGylation is well established. The FDA has authorized 38 PEGylated pharmaceuticals such as PEG-Intron (PEG interferon), Mircera (PEG-epoetin beta), and Doxil (PEGylated liposomal doxorubicin) and 52 currently active clinical trials [13,14]. As a result of exposure to PEG compounds in consumer and pharmaceutical products, anti-PEG antibodies were detected in 0.2% of the healthy population in 1984 [15]. Approximately 3 decades later, Garay et al. [16] reported that the prevalence of pre-existing anti-PEG antibodies had increased to 25%. By 2016, the prevalence had risen to 44.3% among healthy blood donors [17]. A recent study measuring 300 human plasma samples found that anti-PEG immunoglobulin G (IgG) or immunoglobulin M (IgM) was detected in 65.3% of healthy donors [18]. With the heightened sensitivity of detection assays, there has been a significant increase in the detected prevalence of anti-PEG antibodies within the population over the past 4 decades. As people begin receiving multiple doses of Comirnaty, it is critical to ascertain whether anti-PEG antibodies affect the vaccine’s efficacy and safety.

Anti-PEG antibodies are found in animal models, human patients, and even healthy humans [19]. These pre-existing anti-PEG antibodies can contribute to the limiting of therapeutic efficacy. For example, after a single administration of pegloticase, a PEGylated porcine uricase, 38% of patients had elevated anti-PEG antibodies within 3 weeks, resulting in the loss of pegloticase efficacy [20]. Likewise, the accumulation of PEGylated liposomes was 2.7-fold lower in tumors of mice with pre-existing anti-PEG antibodies compared to that in naïve mice. Pre-existing anti-PEG antibodies also reduced the therapeutic efficacy of PEGylated liposomal doxorubicin in vivo [21]. Human anti-PEG IgG triggered complement activation, causing membrane attack complexes to develop in the phospholipid bilayer of liposomes and cause rapid release of doxorubicin from Doxisome [22]. Moreover, anti-PEG IgM can destabilize PEGylated mRNA–LNPs, leading to the release of approximately 34% and 50.5% of mRNA in 5 mol% PEG_2K_–1,2-distearoyl-*sn*-glycero-3-phosphorylethanolamine (DSPE) LNPs and 1.5 mol% PEG_2K_–dimethylglyoxime, respectively. This release occurs through the activation of the complement system, facilitating the liberation of encapsulated mRNA [23]. These findings indicate that anti-PEG antibodies can reduce therapeutic efficacy, alter pharmacokinetics, and potentially destroy the intact membrane of liposomal drugs, leading to accelerated blood clearance. Collectively, it is crucial to study the influence of pre-existing anti-PEG antibodies on PEGylated mRNA vaccines.

We aimed to establish pre-existing anti-PEG antibody mouse models by immunizing mice with PEGylated bovine serum albumin (BSA) and ovalbumin (OVA) to induce the production of anti-PEG antibodies. The mouse models were divided into 3 groups for analysis. Group 1 (G1) had an anti-PEG IgG + IgM concentration ranging from 0.76 to 27.41 μg/ml. Group 2 (G2) had a concentration ranging from 31.27 to 99.52 μg/ml. The naïve group served as the control group, with an anti-PEG IgG + IgM concentration of 0 μg/ml. To begin the experiment, we administered 3 doses of Comirnaty to the pre-existing anti-PEG antibody mouse models to study the correlation between pre-existing anti-PEG antibodies and the response to Comirnaty. The mouse model was also used to compare anti-spike antibody titers among the 3 groups after the mice were administered a total of 3 doses of Comirnaty. We also examined the pharmacokinetics of spike protein production after the 1st vaccine dose. Additionally, we conducted the same protocol on passively transferred anti-PEG antibodies in mouse models to specifically verify that anti-PEG antibodies could reduce the efficacy of the mRNA vaccine. Moreover, to discover whether the PEG molecule is the allergen that can cause anaphylaxis, we detected hypersensitivity indicator complement C3a in this model. We also assessed if anti-PEG antibodies were elicited by 3 doses of Comirnaty. This study examined the relationship between mRNA vaccines and anti-PEG antibodies. The findings could serve as a valuable resource for determining the most appropriate type of vaccine to choose.

## Materials and Methods

### Mice

Specific pathogen-free female BALB/cByJNarl mice aged 4 to 8 weeks were purchased from the National Laboratory Animal Center in Taipei, Taiwan. All mice were maintained according to institutional guidelines and approved by the Animal Care and Use Committee of Kaohsiung Medical University.

### Comirnaty vaccine

Comirnaty was a kind gift from the Central Epidemic Command Center, Taiwan. The vaccine was maintained at 4 °C before use. We reconstituted the Comirnaty mRNA vaccine with 1.8 ml of sterile phosphate-buffered saline (PBS) (pH = 7.4 to 7.5) into the concentrated vaccine. The vaccine stock was diluted to 50 μg/ml before each experiment.

### PEGylated BSA and OVA preparation

Succinimidyl propionic acid PEG (PG1-SPA-2k, Nanocs) and BSA (A7906, Sigma-Aldrich) or OVA (A5503, Sigma-Aldrich) were mixed at a molar ratio of 40:1 in 0.1 M NaHCO_3_, pH = 8.5, for 2 h at room temperature. One-tenth volume of 1 M Tris buffer (pH = 8) was added to stop the reaction. PD-10 columns (17085101, Cytiva) were used to remove free PEG and change the buffer to PBS. Protein concentrations were analyzed (Pierce BCA Protein Assay Kit, Thermo Fisher Scientific) with BSA used as the reference protein. The molecular weights of PEGylated BSA and OVA were detected by sodium dodecyl sulfate polyacrylamide gel electrophoresis (data not shown), and PEG attachment was confirmed by the first anti-PEG antibody (clone 6.3) and the secondary Peroxidase-AffiniPure Goat Anti-Rabbit IgG Fc Fragment Specific (111-035-008, Jackson ImmunoResearch) using Western blot (data not shown).

### Pre-existing and passive transfer anti-PEG antibodies’ in vivo models

**Fig. 1.**
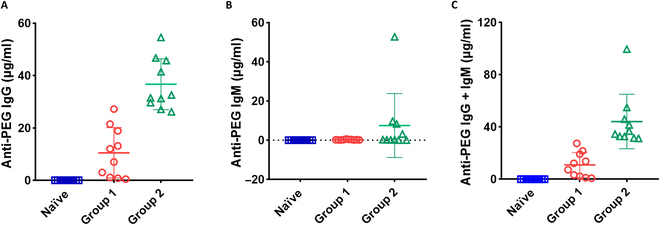
Establishment of a pre-existing anti-polyethylene glycol (anti-PEG) antibody mouse model. Mice were immunized with PEGylated bovine serum albumin (BSA) and ovalbumin (OVA) immunogens to induce the production of anti-PEG antibodies. Plasma was collected from mice following a subcutaneous injection of PEG_2K_–BSA followed by a subcutaneous injection of PEG_2K_–OVA 1 month later. Concentrations of (A) anti-PEG immunoglobulin G (IgG), (B) immunoglobulin M (IgM), and (C) IgG + IgM were measured in the naïve BALB/cByJNarl mice (□) (*n* = 10), group 1 (◯) (*n* = 10), and group 2 (△) (*n* = 10) by enzyme-linked immunosorbent assay (ELISA).

The 2 pre-existing anti-PEG antibody mouse models, the pre-existing anti-PEG antibody model and the passive transfer anti-PEG antibody model, were generated following previously established protocols. The pre-existing anti-PEG antibody mouse model (*n* = 35) was established according to the conventional protocol developed by Leenaars and Hendriksen [24]. We prepared Freund’s adjuvants and combined approximately equal volumes of the adjuvant with either PEG_2K_–OVA or PEG_2K_–BSA as water-in-oil emulsions. We changed different species of proteins to prevent mice from producing large amounts of anti-OVA or anti-BSA antibodies rather than anti-PEG antibodies. BALB/cByJNarl mice were subcutaneously injected with emulsions of PEG_2K_–BSA with Freund’s complete adjuvants (F5881, Sigma) as the first immunogen and emulsions of PEG_2K_–OVA with Freund’s incomplete adjuvants (F5506, Sigma) as the second immunogen. After 2 doses of immunization, indirect enzyme-linked immunosorbent assay (ELISA) was performed to detect the concentration of anti-PEG antibodies in mouse plasma. We used standard anti-PEG IgG (clone 6.3) and anti-PEG IgM (clone AGP_4_) serial dilutions for standard curves to interpolate the concentration of anti-PEG antibodies in mouse plasma. The groups were divided based on the concentration of anti-PEG IgG and IgM antibodies (Fig. 1). The passive transfer anti-PEG antibody model (Fig. S1) was established by intravenously injecting different concentrations of mouse anti-PEG IgG (clone 6.3) or IgM (clone AGP_4_) antibody; after 4 h, blood was drawn to confirm the concentration of anti-PEG antibodies in mice [21].

### Measurement of pre-existing anti-PEG antibodies by indirect ELISA

ELISA plates (Clear Flat-Bottom Immuno Nonsterile 96-Well Plates, Thermo Fisher Scientific) were coated with 20 μg/ml methyl–PEG_2K_–NH_2_ in 0.1 M NaHCO_3_, pH = 9, at 4 °C overnight. The following day, the plates were blocked with 5% skim milk in PBS (pH = 7.4 to 7.5) for 1 h at room temperature. The plates were washed with PBS 3 times and blotted with a paper towel. Mouse plasma samples and standard anti-PEG antibodies 6.3 (IgG) and AGP_4_ (IgM) were serially 2-fold diluted from 0.5 to 0.0039 μg/ml and added to predesignated wells (50 μl/well) for 1 h at room temperature. After washing with PBS 3 times, horseradish peroxidase-conjugated goat antimouse IgG, Fcγ fragment specific (115-035-008, Jackson ImmunoResearch), and goat antimouse IgM, μ chain specific (115-035-020, Jackson ImmunoResearch), were added to the wells. The plates were washed with PBS 3 times, and the bound antibody was measured by adding 150 μl/well 2,2′-azino-bis(3-ethylbenzothiazoline-6-sulfonic acid) diammonium salt (ABTS) solution (0.003% H_2_O_2_ and 100 mM phosphate–citrate, pH = 4.0). The absorbance was measured at OD_405nm_ with blank subtraction. All incubations were conducted in duplicate, and data are presented as mean concentrations.

### Assessment of the amount of anti-spike antibody in mouse plasma

The mice were injected with 20 μl of Comirnaty (50 μg/ml) intramuscularly. Each dose contained 1 μg of mRNA. We administered 3 vaccinations in total, 1 month apart. Tail vein samples were collected using heparinized microhematocrit capillaries (Hecht Assistant, 40563) on days 7, 14, and 28. Plasma was separated by centrifugation at 3,000 rpm for 10 min at 22 to 25 °C. To detect anti-spike antibodies, 96-well plates (442404, Clear Flat-Bottom Immuno Nonsterile 96-Well Plates, Thermo Fisher Scientific) were coated with recombinant S1 100 ng/100 μl in PBS, pH = 7.4 (catalog no.: 40591-V08H; Sino Biological, Beijing, China) for 2 h at 37 °C. Diluted mouse plasma samples were added into the plate (50 μl/well) and incubated for 50 min at room temperature. Bound mouse anti-spike antibodies were detected using horseradish peroxidase-conjugated goat antimouse IgG + IgM (H + L) (115-035-044, Jackson ImmunoResearch). The plates were washed with PBS with Tween 20 (pH = 7.4 to 7.5) 3 times and PBS (pH = 7.4 to 7.5) once, and the bound antibodies were measured by adding 150 μl/well ABTS solution (0.003% H_2_O_2_ and 100 mM phosphate–citrate, pH = 4.0). The absorbance was measured at OD_405nm_, and the data represent the value divided by untreated control. All incubations were conducted in duplicate, and data are presented as means.

### Quantitative determination of mouse complement C3a concentrations in plasma

Mouse blood was collected after vaccination at 15 min, 3 h, 8 h, and 24 h. The complement C3a concentration in 50-fold diluted mouse plasma was measured using Mouse Complement C3a ELISA Kit (NBP2-70037, Novus Biologicals) according to the manufacturer’s instructions.

### Plasma SARS-CoV-2 spike protein measurement

Plasma concentrations of severe acute respiratory syndrome coronavirus 2 (SARS-CoV-2) spike protein were measured using SARS-CoV-2 Spike Protein Titer Assay Kit (RAS-A020, ACROBiosystems). Mouse blood was collected at 15 min, 3 h, 8 h, 24 h, 48 h, 72 h, and 168 h after vaccination; 40-, 80-, and 160-fold diluted mouse plasmas were assayed according to the manufacturer’s instructions. Using the standards provided by the kit as reference, the levels of SARS-CoV-2 spike protein were interpolated using the value of OD_630nm_ subtracted from OD_450nm_.

### Statistical analysis

**Fig. 2.**
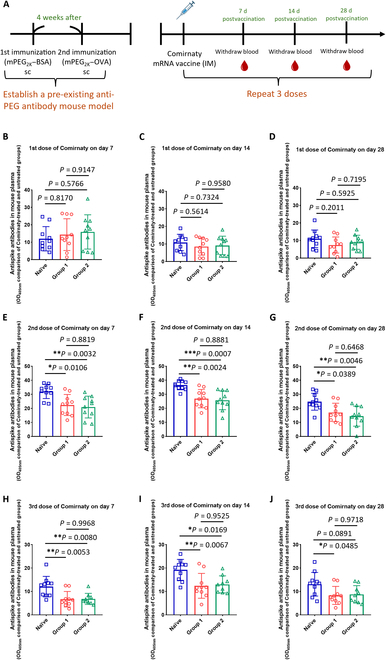
Impact of pre-existing anti-PEG antibody levels on the efficacy of anti-spike protein antibodies in vivo. Group 1 (G1), group 2 (G2), and naïve mice received 3 doses of Comirnaty, each containing 1 μg of messenger RNA (mRNA), administered intramuscularly. We examined mouse plasma samples to detect anti-spike antibodies at 7, 14, and 28 d following each vaccination. (A) Schedule of experiments. (B to D) Comparison of anti-spike antibodies in mouse plasma (1,000-fold dilution) among the naïve (□), G1 (◯), and G2 (△) mice by the absorbance of OD_405nm_ after the 1st vaccination on days 7, 14, and 28 (naïve group, G1, and G2, all *n* = 10). (E to G) Comparison of anti-spike antibodies in mouse plasma (10,000-fold dilution) among the naïve, G1, and G2 mice by the ELISA absorbance of OD_405nm_ after the 2nd vaccination on days 7, 14 (naïve, G1, and G2, all *n* = 10), and 28 (naïve group and G1, *n* = 10; G2, *n* = 9). (H to J) Comparison of anti-spike antibodies in mouse plasma (50,000-fold dilution) among the naïve, G1, and G2 mice by the ELISA absorbance of OD_405nm_ after the 3rd dose vaccination on days 7, 14, and 28 (naïve and G1, *n* = 10; G2, *n* = 9). Statistical analyses were conducted using one-way analysis of variance (ANOVA) followed by Tukey’s multiple comparisons test. Asterisks denote significance levels: *P < 0.05, **P < 0.01, and *** *P* < 0.001 compared to the naïve group. sc, subcutaneous; IM, intramuscular.

**Fig. 3.**
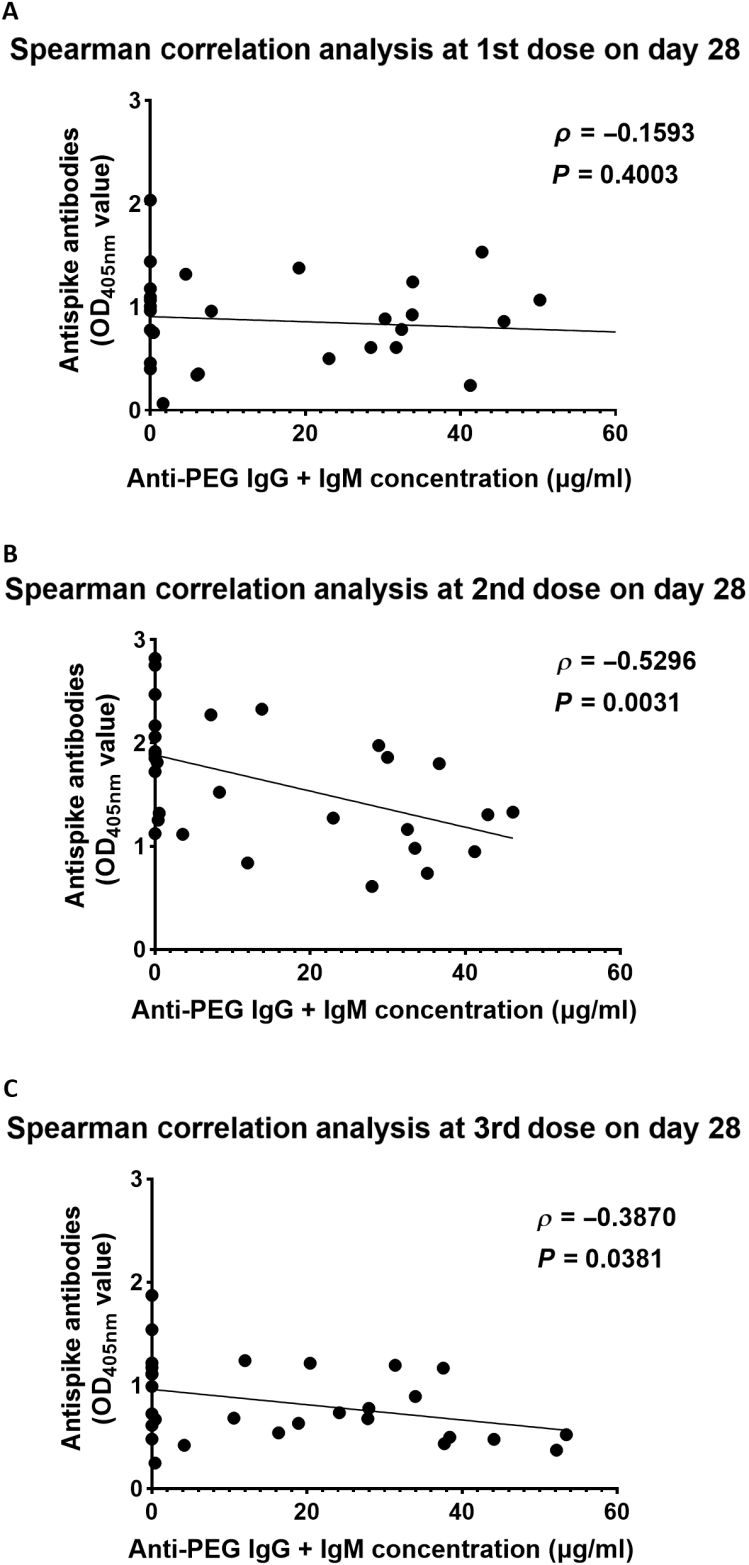
Spearman’s rank correlation between anti-spike and anti-PEG antibodies in mouse plasma after 3 doses of Comirnaty. We conducted a Spearman’s rank correlation analysis to examine the relationship between the absorbance at OD_405nm_ of anti-spike antibodies and the concentration of anti-PEG IgG + IgM antibodies. (A) The data were analyzed on day 28 after administering the 1st vaccine dose. (B) Data analysis was conducted on day 28 after the 2nd vaccine dose. (C) The data were analyzed on day 28 after the 3rd vaccine dose. Spearman’s rank correlation coefficient (*ρ*) was calculated, and statistical significance was determined. A *P* value of less than 0.05 was considered statistically significant.

**Fig. 4.**
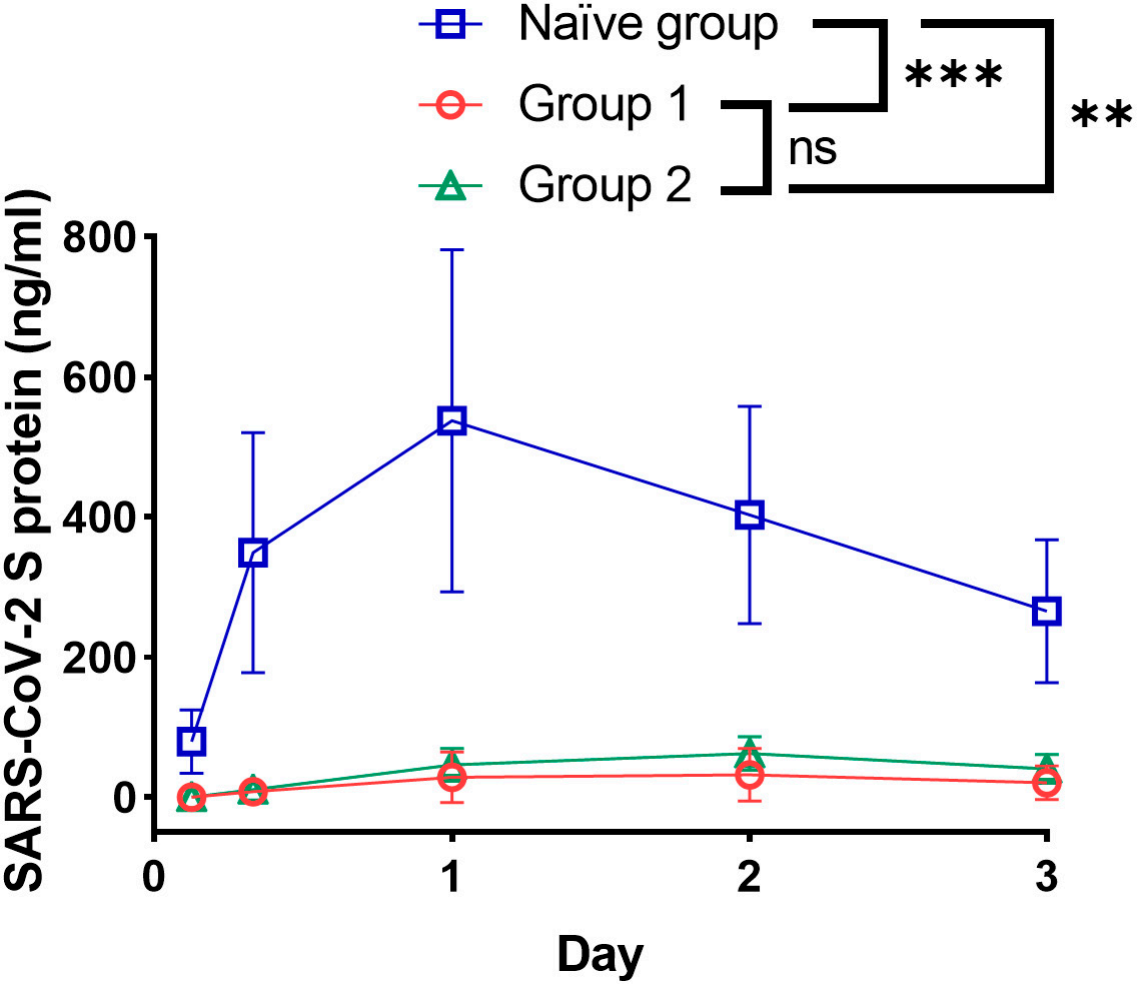
Impact of pre-existing anti-PEG antibodies on the concentration of spike protein in mice. The mice were then given an intramuscular injection of Comirnaty, containing 1 μg of mRNA. All mice were bled at 3, 8, 24, 48, and 72 h. The plasma was diluted 40-fold in the diluent buffer provided by the severe acute respiratory syndrome coronavirus 2 (SARS-CoV-2) spike protein kit. We used this kit to measure the levels of spike protein (ng/ml) in the naïve, group 1, and group 2 mice (all groups, *n* = 3). Statistical analysis was conducted using ordinary one-way ANOVA followed by Tukey’s multiple comparisons test. Statistical significance was indicated as follows: * *P* < 0.05, **P < 0.01, and ***P < 0.001 compared to the naïve group. ns, not significant.

**Fig. 5.**
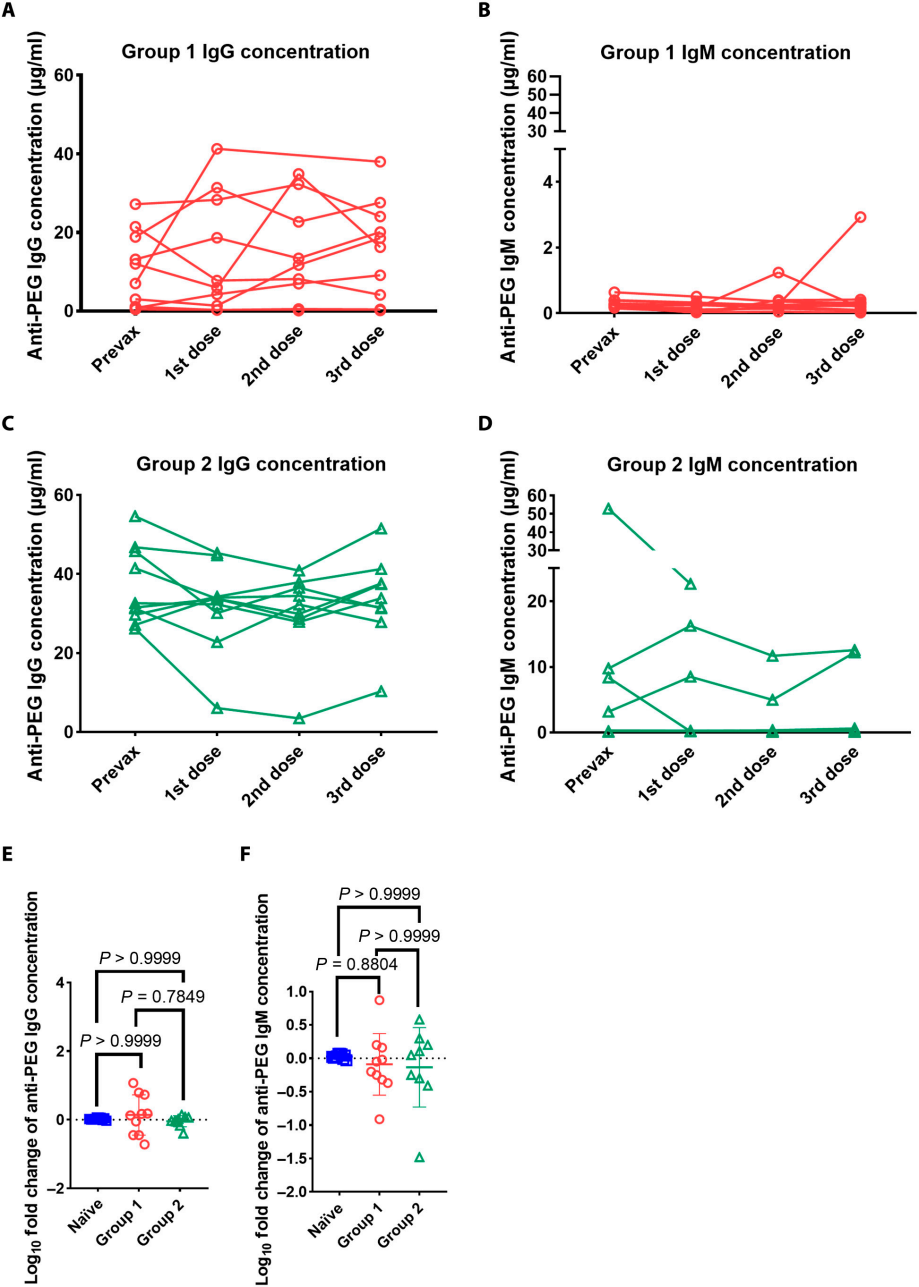
Repeat injection of Comirnaty marginally induced PEG-specific antibodies. The concentration of anti-PEG antibodies in mouse plasma was assessed on day 28 after each vaccination. (A) A comparison of the concentrations of anti-PEG IgG before vaccination and after 3 doses of Comirnaty in group 1 (◯) (*n* = 10). (B) A comparison of the concentrations of anti-PEG IgM before vaccination and after 3 doses of Comirnaty in group 1 (◯) (*n* = 10). (C) A comparison of the concentrations of anti-PEG IgG before vaccination and after 3 doses of Comirnaty in group 2 (△) (*n* = 10). (D) A comparison of the concentration of anti-PEG IgM before vaccination and after 3 doses of Comirnaty in group 2 (△) (prevax and 1st dose, *n* = 10; 2nd dose and 3rd dose, *n* = 9). (E) Cross-comparison of the fold-change (log_10_) of anti-PEG IgG and IgM (F) among the 3 cohorts: naïve groups (prevax/3rd dose, *n* = 10), group 1 (prevax/3rd dose, *n* = 10), and group 2 (prevax/3rd dose, *n* = 9). (E and F) Data were derived by the nonparametric Kruskal–Wallis test with Dunn’s multiple comparisons test (*n* = 10 in naïve group and group 1; *n* = 9 in group 2). Prevax, prevaccination; 3rd dose, 3rd dose of Comirnaty vaccination.

**Fig. 6.**
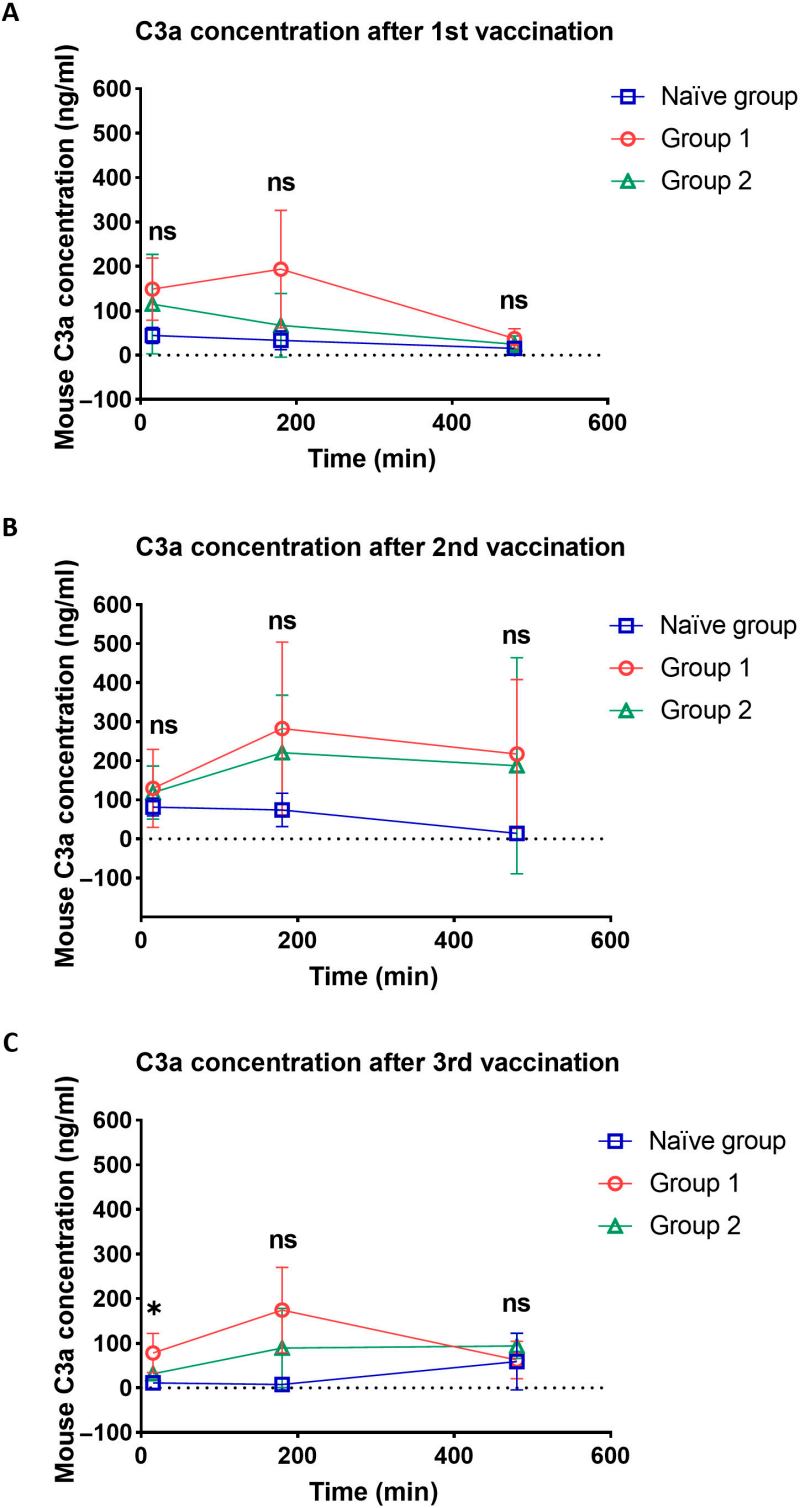
Pre-existing anti-PEG antibody release of complement C3a in mouse plasma. We analyzed C3a levels using a commercial ELISA kit after the 1st (A), 2nd (B), and 3rd (C) doses of Comirnaty. C3a concentrations were measured across the 3 groups at 15 min, 3 h, and 8 h. Statistical comparisons between groups were conducted using 2-way ANOVA, followed by Tukey’s multiple comparisons test. Statistical significance is indicated as follows: *P < 0.05, **P < 0.01, and ***P < 0.001, with group 2 compared to the naïve group. Each sample was measured in duplicate (*n* = 3).

Statistical significance in Fig. 2B to J was assessed using ordinary one-way analysis of variance (ANOVA), followed by Tukey’s multiple comparisons test, conducted with GraphPad Prism 8.0. Correlation analysis in Fig. 3A to C were performed using nonparametric Spearman correlation tests, also using GraphPad Prism 8.0. Figure 4 was assessed using ordinary one-way ANOVA, followed by Tukey’s multiple comparisons test, conducted with GraphPad Prism 8.0. Cross-comparisons among the 3 groups in Fig. 5E and F were conducted with the nonparametric Kruskal–Wallis test, followed by Dunn’s multiple comparisons test. The statistical significance in Fig. 6A to C was analyzed using 2-way ANOVA with Tukey’s multiple comparisons test. Asterisks indicating statistical significance between groups are defined as *P < 0.05, **P < 0.01, and ***P < 0.001.

## Results

### Establishment of a pre-existing anti-PEG antibody mouse model

To imitate the wide range of pre-existing anti-PEG antibodies found in humans, we initially created a mouse model with pre-existing anti-PEG antibodies. We achieved this by subcutaneously injecting PEG_2K_–BSA into naïve BALB/cByJNarl mice, followed by an injection of PEG_2K_–OVA 1 month later. Employing various protein species, we sought to inhibit the mice from generating substantial quantities of anti-OVA or anti-BSA antibodies, thereby maintaining the emphasis on eliciting anti-PEG antibodies. The concentration of anti-PEG IgG or IgM antibody in plasma samples was measured by indirect ELISA in 96-well plates coated with 2,000-Da methyl PEG. The IgG concentration ranged from 0.46 to 54.6 μg/ml. The IgM concentration ranged from 0.15 to 52.77 μg/ml (Table). We separated the groups according to Deuker et al., who evaluated the prevalence of anti-PEG antibodies in 500 healthy donors prior to the approval and administration of PEGylated COVID-19 vaccines. Their study indicated that the mean concentration of anti-PEG IgG was approximately 20 to 30 μg/ml [25]. Given that individuals who have received PEGylated pharmaceuticals may develop higher levels of anti-PEG antibodies, we established a threshold of 30 μg/ml to distinguish between the groups. To investigate how pre-existing anti-PEG antibodies in healthy donors and individuals who received PEGylated pharmaceuticals affect the efficacy of the mRNA vaccine, we divided the mice into 2 groups according to this threshold. Figure 1A and B show the concentrations of anti-PEG IgG and IgM antibodies in the 3 groups: G1 (*n* = 10), G2 (*n* = 10), and an additional group of naïve mice (*n* = 10) that were not immunized. In G1, the concentrations of anti-PEG IgG ranged from 0.46 to 27.22 μg/ml, while the concentrations of anti-PEG IgM ranged from 0.15 to 0.64 μg/ml. In G2, the concentrations of anti-PEG IgG and IgM ranged from 26.16 to 54.6 μg/ml and from 0.1 to 52.77 μg/ml, respectively. The concentration range of anti-PEG IgG + IgM in G1 was 0.76 to 27.41 μg/ml, and in G2, it was 31.27 to 99.52 μg/ml (Fig. 1C). Based on our results, the concentration of the pre-existing anti-PEG antibody in our mouse model is comparable to that found in the human population [17,18]. The results demonstrated that we effectively created a mouse model with pre-existing anti-PEG antibodies that closely mirror those of humans.

**Table.** The concentrations of anti-PEG IgG and IgM in each mouse in the pre-existing anti-PEG antibody mouse model

Naïve group (*n* = 10)											
Ear punching identification no.	13-UN	13-L1	13-R1	13-L2	13-LR	14-UN	14-L1	14-R1	14-L2	14-LR	Average concentration (μg/ml)
Anti-PEG IgG concentration (μg/ml)	0.00	0.00	0.00	0.00	0.00	0.00	0.00	0.00	0.00	0.00	0.00
Anti-PEG IgM concentration (μg/ml)	0.00	0.00	0.00	0.00	0.00	0.00	0.00	0.00	0.00	0.00	0.00
Anti-PEG IgG + IgM concentration (μg/ml)	0.00	0.00	0.00	0.00	0.00	0.00	0.00	0.00	0.00	0.00	0.00
Group 1 (*n* = 10)											
Ear punching identification no.	2-L1	8-UN	7-R1	12-R1	5-UN	4-L1	3-R1	11-UN	5-R1	9-R1	Average concentration (μg/ml)
Anti-PEG IgG concentration (μg/ml)	1.13	3.04	7.02	0.46	0.78	13.14	21.47	12.03	18.88	27.22	10.52
Anti-PEG IgM concentration (μg/ml)	0.17	0.23	0.27	0.30	0.39	0.64	0.18	0.15	0.40	0.19	0.29
Anti-PEG IgG + IgM concentration (μg/ml)	2.30	3.27	7.29	0.76	1.17	13.78	21.65	12.18	19.28	27.41	10.81
Group 2 (*n* = 10)											
Ear punching identification no.	4-UN	10-R1	10-L1	10-UN	11-L1	9-UN	12-L1	6-L1	11-R1	2-R1	Average concentration (μg/ml)
Anti-PEG IgG concentration (μg/ml)	45.77	41.46	32.64	31.51	54.60	31.28	26.16	46.75	27.13	29.67	36.70
Anti-PEG IgM concentration (μg/ml)	0.19	0.10	0.12	0.21	0.31	0.30	8.36	52.77	9.74	3.18	7.53
Anti-PEG IgG + IgM concentration (μg/ml)	45.96	41.56	32.76	31.72	54.91	31.58	34.52	99.52	36.87	32.85	44.23

### Impact of pre-existing anti-PEG antibodies on the efficacy of the COVID-19 mRNA vaccine in mice

Several studies have shown that pre-existing anti-PEG antibodies impact the stability, pharmacokinetics, and efficacy of PEGylated liposomal drugs [23,26]. To investigate the potential impact of pre-existing antibodies on the effectiveness of Comirnaty, we administered 3 doses of Comirnaty via intramuscular injection to naïve, G1, and G2 mice. The mice were injected with 20 μl of Comirnaty (50 μg/ml) intramuscularly. Each dose contained 1 μg of mRNA. A total of 3 vaccinations were given, with a 1-month interval between each vaccine dose. The mouse plasmas of the 3 groups were assessed on days 7, 14, and 28 after each vaccination event (Fig. 2A). The first set of analyses examined the impact of anti-PEG antibodies on the efficacy of Comirnaty. There was no significant difference in the OD_405nm_ values of anti-spike antibodies among the 3 groups after the 1st vaccination (Fig. 2B to D). After the 2nd dose, the OD_405nm_ values of anti-spike antibodies were significantly reduced in both G1 (day 7, *P* = 0.0106; day 14, *P* = 0.0024; and day 28, *P* = 0.0389) and G2 (day 7, *P* = 0.0032; day 14, *P* = 0.0007; and day 28, *P* = 0.0046) as compared to those in the naïve group mice (Fig. 2E to G). The OD_405nm_ values of anti-spike in the PEG antibody groups were also significantly lower after the 3rd vaccination (G1: day 7, *P* = 0.0053; day 14, *P* = 0.0067; and day 28, *P* = 0.0485) (G2: day 7, *P* = 0.0080; day 14, *P* = 0.0169; and day 28, *P* = 0.0891) (Fig. 2H to J). These results show that pre-existing anti-PEG antibodies can reduce the efficacy of Comirnaty.

We analyzed possible correlations between pre-existing anti-PEG antibodies and anti-spike antibodies on day 28 after each of the 3 vaccine immunizations. Spearman’s rank correlation analysis showed no significant correlation between the concentration of pre-existing anti-PEG antibodies and anti-spike antibodies on day 28 after the 1st vaccination (1st dose: *ρ* = −0.1593, *P* = 0.4003) (Fig. 3A). However, there was a significant negative correlation between pre-existing anti-PEG antibodies and anti-spike antibodies after the 2nd and 3rd vaccine doses (2nd dose: *ρ* = −0.5296, *P* = 0.0031; 3rd dose: *ρ* = −0.3870, *P* = 0.0381) (Fig. 3B and C). These results indicate that higher concentrations of anti-PEG antibodies are associated with a greater reduction in the OD_405nm_ values of anti-spike antibodies.

To distinguish the effect between the isotypes of anti-PEG antibodies, we further establish a passively transfer anti-PEG antibody mouse model to confirm the influence of IgG and IgM. Closer inspection of Fig. S1 shows that anti-PEG IgG antibodies (clone 6.3) rather than IgM (clone AGP_4_) had a dominant effect on Comirnaty.

### Impact of pre-existing anti-PEG antibodies on the concentration of spike protein in mice

While the mRNA of Comirnaty is designed to encode the spike protein in its prefusion form, the full-length spike protein may either anchor on the cell surface, be secreted extracellularly, or undergo dissociation of the spike 1 protein through furin cleavage within the trans-Golgi network [27]. To investigate the potential impact of pre-existing anti-PEG antibodies on spike protein pharmacokinetics, we injected a single dose of Comirnaty into the naïve, G1, and G2 mice and then monitored the fluctuations in spike protein levels in their plasma. We collected mouse plasma at 3, 8, 24, 48, and 72 h after vaccination and employed a sandwich ELISA kit to measure the amount of spike protein. Notably, the concentration of spike protein in the naïve group was 45.4-fold higher than that in G1 and 34.1-fold higher than that in G2 at 8 h. One-way ANOVA revealed a significantly reduced level of spike protein in plasma samples in G1 and G2 mice as compared to the naïve group with *P* values of 0.001 and 0.014, respectively (Fig. 4). These results indicate that pre-existing anti-PEG antibodies diminished the amount of spike protein in plasma and suggested that they also inhibit production of spike protein.

### Levels of anti-PEG antibodies after 3 doses of Comirnaty

To determine whether Comirnaty vaccination can induce the production of anti-PEG antibodies, we measured anti-PEG antibody levels in mouse plasma samples from the naïve, G1, and G2 mice using indirect ELISA on day 28 after each vaccine administration. To prevent anti-PEG antibodies from neutralizing subsequently injected PEGylated mRNA vaccines and inducing an anti-PEG antibody sink effect, which could interfere with the accurate quantification of antibody levels, we opted to conduct the detection on day 28. Figure 5A and B show a marginal elevation in the level of anti-PEG IgG in G1. There was an increase in the average concentration of anti-PEG IgG from 10.52 to 15.83 μg/ml and an increase in the average concentration of anti-PEG IgM from 0.29 to 0.49 μg/ml. Figure 5C and D indicate that there is only a slight alteration of anti-PEG antibodies in G2. The mean concentration of anti-PEG IgG exhibited a minimal decrease from 36.7 to 33.64 μg/ml in G2. The mean concentration of anti-PEG IgM decreased insignificantly from 7.53 to 2.92 μg/ml in G2. We further examined the fold change of antibody levels against PEG in the 3 different groups. Comparing the fold changes of anti-PEG antibody levels across the 3 groups (Fig. 5E and F), we conclude that both anti-PEG IgG and IgM were not significantly boosted by Comirnaty vaccination in mice. The average fold changes of anti-PEG IgG and IgM antibodies in the naïve group, G1, and G2 were 0.03-fold, 0.14-fold, and −0.04-fold and 0.03-fold, −0.09-fold, and −0.13-fold, respectively, as shown in Fig. 5E and F. Overall, these results suggest that a total of 3 doses of Comirnaty did not significantly lead to an efficient boost of anti-PEG antibodies in our mouse model.

### Concentrations of complement C3a in Comirnaty-immunized mice

To ascertain the potential allergenicity of Comirnaty in mice with anti-PEG antibodies, we measured the levels of C3a, an anaphylatoxin, as an indicator [28,29]. We administered 3 doses of Comirnaty to the naïve, G1, and G2 mice and monitored the concentration of mouse complement C3a at 15 min, 3 h, and 8 h postadministration. The mean concentrations of C3a in G1 were 149, 129, and 78 ng/ml, respectively, while in Group 2, the concentrations were 115, 119, and 32 ng/ml. In the naïve group, the concentrations were 44, 82, and 11 ng/ml at the same time points (Fig. 6A to C). Notably, a significantly higher concentration of C3a was observed in G2 compared to that in the naïve group 15 min postvaccination after the 3rd dose (*P* = 0.0483) (Fig. 6C). We also observed 16% (4 of the 25) pre-existing anti-PEG antibody mice displayed alopecia (Fig. S2). Of note, alopecia areata has also been reported in the clinic. For example, a 37-year-old woman experienced low-grade fever post her 1st Comirnaty dose and had self-detected coin-sized hair loss, which was diagnosed as alopecia areata. Skin biopsy revealed immune cell infiltration and increased telogen hairs. An Italian case report showed similar hair loss under trichoscopy with no autoantibodies detected. Whether mRNA vaccines trigger immune responses harming hair follicles or magnify through anti-PEG antibodies requires further study [30–32].

## Discussion

**Fig. 7.**
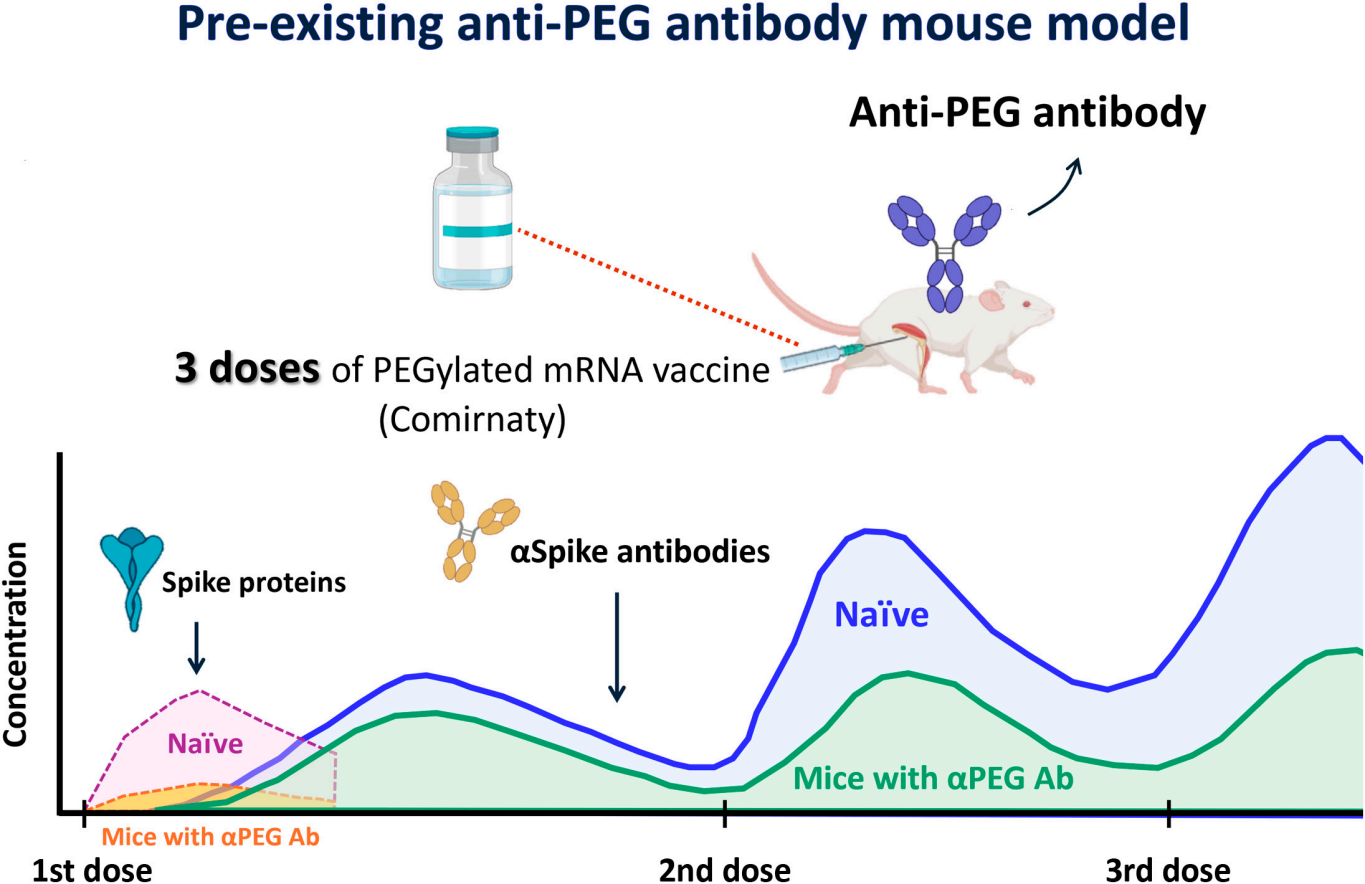
Impact of pre-existing anti-PEG antibodies on the pharmacokinetics and efficacy of the COVID-19 mRNA vaccine (Comirnaty) in vivo*.* We established a mouse model with pre-existing anti-PEG antibodies and administered a total of 3 doses of Comirnaty. In this model, mice with pre-existing anti-PEG antibodies exhibited significantly reduced spike protein production compared to the naïve group after the 1st dose. Following the 2nd dose, there was a notable decrease in plasma anti-spike antibody concentrations in the pre-existing anti-PEG antibody group compared to those in the naïve group. These findings highlight the impact of anti-PEG antibodies on the efficacy of PEGylated mRNA vaccines. Therefore, individuals with pre-existing anti-PEG antibodies are advised to consider vaccines that do not contain PEG. αPEG Ab, anti-PEG antibody.

Our research investigated the impact of pre-existing anti-PEG antibodies in a mouse model to elucidate the interaction between these antibodies and Comirnaty. We found that pre-existing anti-PEG antibodies significantly reduce the potency of Comirnaty. In addition, pre-existing anti-PEG antibodies also reduced the concentration of spike protein in a pre-existing anti-PEG antibody mouse model. We noted a significantly increased concentration of complement C3a in G2 mice following the 3rd vaccine dose, compared to that in the naïve group. Evidence from this study suggests that pre-existing anti-PEG antibodies generate a negative effect on the PEGylated mRNA vaccine (Fig. 7). mRNA-based technology is expected to impact not only vaccinology but also cancer immunotherapy, therapeutic protein replacement therapies, and genetic disorder treatment [5]. A well-established mouse model that generates antibodies against PEG can expedite the advancement of mRNA-based technology. This model is significant because it faithfully reproduces the diverse spectrum of anti-PEG antibody titers observed in human populations. This study reveals potential issues in which pre-existing anti-PEG antibodies might impact individuals who receive PEGylated mRNA vaccines and might reduce the therapeutic effect of PEGylated mRNA-based technology.

Several studies have examined the influence of anti-PEG antibodies on COVID-19 mRNA vaccines [26,33–35]. However, due to differences in the size of the cohorts, ages, sex, geography, and the sample collection time points in the human subjects, it is still controversial to presume whether anti-PEG antibodies can affect the efficacy of PEGylated mRNA vaccines. For example, Ju et al. [36] reported that anti-PEG IgM and IgG concentrations significantly increased by a mean of 1.78-fold and 2.64-fold, respectively, after 2 doses of Comirnaty. However, they did not observe any correlation between anti-PEG antibody responses and vaccine effectiveness. Carreño et al. [37] did not find any changes in the levels of anti-PEG antibodies after 1 or 2 doses of Comirnaty, whereas those who received the mRNA-1273 vaccine showed an increase in antibodies that specifically targeted PEG-BSA. However, Bavli et al. [34] uncovered a significant rise in the mean concentration of anti-PEG IgG antibodies, increasing from 7.8 to 17.5 μg/ml, while there was no observed significant increase in the IgM subtype. We found only a negligible change in the concentration of anti-PEG IgG and IgM antibodies in G1 (0.14-fold and −0.09-fold changes) and G2 (−0.04-fold and −0.13-fold changes) compared to that prevaccination. Anti-PEG antibodies were not induced after a total of 3 doses of mRNA vaccines in the control group; this result corresponded to that of Carreño et al. Additionally, the concentration of anti-spike antibodies significantly decreased in G1 and G2 mice compared to that in the naïve group. Based on the above results, by using the pre-existing anti-PEG antibody mouse models, we can study the interaction between pre-existing anti-PEG antibodies and PEGylated mRNA vaccines or any PEGylated drugs in depth and objectively, ultimately identifying how pre-existing anti-PEG antibodies affect the vaccine–immune interaction or PEGylated drugs.

Alternative methods for generating mouse models with anti-PEG antibodies have been reported, including the intravenous transfer of mouse anti-PEG antibodies and the transplantation of hybridoma cells. For instance, Lin et al. [38] intravenously administered different PEG IgG or IgM antibodies to assess the relationship between the antibody binding epitope and the clearance of PEGylated compounds. El Sayed et al. [39] investigated the potential of anti-PEG antibodies to reduce the therapeutic efficacy of Doxil through the intraperitoneal inoculation of hybridoma cells (HIK-M09 and HIK-M11), which produce monoclonal anti-PEG IgM [39]. Both methods facilitate direct assessment of the effects of IgG or IgM subtypes on PEGylated pharmaceuticals. However, these approaches yield only monoclonal IgG or IgM antibodies, which do not accurately mimic human immune responses due to the absence of antibody class switching with repeated injections. Additionally, the introduction of hybridoma cells into the peritoneal cavity introduces variability by increasing the risk of cancer development over time. Conversely, we developed an animal model with pre-existing anti-PEG antibodies through immunization with PEG-conjugated proteins. A similar approach involves the intravenous administration of PEGylated pharmaceuticals to stimulate the production of anti-PEG antibodies in mice. For example, Li et al. [40] examined whether pretreatment with high-molecular-weight free PEG could preserve the therapeutic efficacy of pegloticase, finding that pegloticase administration induced the rapid production of approximately 14.8 μg/ml anti-PEG IgG and 3.6 μg/ml anti-PEG IgM in mice. Wang et al. [26] administered 3 doses of Comirnaty to evaluate PEG-related immune responses, specifically the profile of LNP-induced anti-PEG antibodies and the biodistribution of DiR-LNPs following multiple injections. These examples, involving immunization with PEGylated proteins or drugs, can generate polyclonal antibodies and develop immunological memory. The immunization process in these models closely mimics human responses, where exposure to PEGylated proteins or drugs can elicit anti-PEG antibody responses through either the classical T-cell-dependent or T-cell-independent pathways [1]. Moreover, these models enable the study of immune responses following repeated exposure to PEGylated substances, including antibody class switching, immune complex formation, and complement activation. When the immune mechanism in the model closely resembles that of humans, the results possess greater potential for clinical translation. Understanding these factors is crucial for evaluating both the therapeutic limitations and safety risks associated with PEGylated pharmaceuticals in patients with pre-existing anti-PEG antibodies. Consequently, utilizing a mouse model immunized with PEGylated proteins or drugs is more comprehensive than previous immunization methods for assessing the influence of pre-existing anti-PEG antibodies on the efficacy and safety of PEGylated pharmaceuticals.

We reported that anti-PEG IgG antibodies slightly increased (0.14-fold change) in G1, and there was no apparent change (−0.04-fold change) in G2 mice. Moreover, 3 doses of Comirnaty did not induce anti-PEG antibodies in the naïve mice. These results are consistent with those of Carreño et al., who did not detect a significant increase in anti-PEG antibodies in Comirnaty recipients. From our perspective, there are 3 potential explanations for this situation: firstly, the concentration of the PEG-lipid of Comirnaty is around 2,000 times less than that of traditional PEGylated liposomal drugs (Doxil) (1.6 μg/ml versus 3.19 mg/ml) [41], so the recipients vaccinated with Comirnaty elicited subtle anti-PEG antibodies. Secondly, compared to DSPE–PEG_2K_ (C18), which can firmly anchor on the LNPs, the short PEG_2K_–lipid (C14) ALC-0159 rapidly dissociates to become free PEG as a nonprotein antigen in the circulation system, thereby reducing the immunogenicity and immunogen uptake [42]. Additionally, following Comirnaty vaccination, PEG-specific B cells are capable of recognizing PEGylated LNPs, internalizing them, and subsequently translating the mRNA of the spike protein. This translation results in the presentation of spike peptides through the major histocompatibility complex class I [27]. Following that, PEG-specific B cells presenting spike peptides are eliminated by cytotoxic T cells. This process is similar to the infection of B cells by the COVID-19 virus. In the end, anti-PEG antibodies diminished in the circulatory system as a result of the number of PEG-specific B cells decreased. Together, these factors might contribute to the reasons why we could not detect increased levels of anti-PEG antibodies in our mouse model after 3 doses of Comirnaty.

One crucial finding is the concentration of spike protein shrunk in mice with pre-existing anti-PEG antibodies. This intriguing result might be explained by antibody-mediated complement activation and accelerated blood clearance. Antibody binding and complement activation can result in nanoparticle opsonization, LNP destabilization, and altered biodistribution that reduces the production of spike protein. For example, humanized anti-PEG antibodies can trigger complement activation and cause accelerated release of doxorubicin from Doxisome and Doxil; additionally, LNP degradation as observed in cryo-electron microscopy images showed an intact membrane [22]. In our study, we detected a higher concentration of complement C3a and lower production of spike protein in mice with anti-PEG antibodies. Complement activation may expose mRNA for more rapid degradation by RNase.

PEGylation technology is widely applied in medications, processed foods, and cosmetics. As a result of the frequent exposure to PEG-containing products, an increasing number of healthy individuals have pre-existing anti-PEG antibodies before receiving PEGylated drugs or PEGylated mRNA vaccines. However, one of the most significant findings from this study is that pre-existing anti-PEG antibodies can reduce the efficacy of multiple doses of mRNA vaccines. To address this issue in the long term, it is crucial to explore alternative PEG molecules, such as poly(glycerols), biocompatible poly(*N*-vinylpyrrolidone), and highly hydrophilic poly(carboxybetaine) [43], for potential pharmaceutical applications. While these alternative PEGylation strategies show promise, further clinical trials are needed to evaluate their safety. Presently, developing diagnostic tests to detect pre-existing anti-PEG antibodies is crucial for identifying individuals who may not be suitable candidates for PEGylated vaccines. Those who test positive for anti-PEG antibodies could be offered alternatives, such as adenovirus vector vaccines like the Oxford–AstraZeneca and Johnson & Johnson COVID-19 vaccines or protein-based vaccines such as MVC-COV1901 [44,45]. Implementing both long-term and short-term strategies will facilitate the development of the next generation of mRNA–LNP technology and enhance safety in pharmaceutical applications.

## Data Availability

All data presented in this manuscript, including supplementary information, are available from the corresponding authors upon reasonable request.
