# Supplementary material for: Impact of Pre-existing Anti-polyethylene Glycol Antibodies on the Pharmacokinetics and Efficacy of a COVID-19 mRNA Vaccine (Comirnaty) In Vivo
Source: Biomater Res. 2024 Dec 11;28:0112. doi: 10.34133/bmr.0112 (PMC11633857; doi:10.34133/bmr.0112)
Supplement: Supplementary 1 — Table S1 Figs. S1 and S2 [file bmr.0112.f1.docx]

**Impact of Pre-Existing Anti-Polyethylene Glycol Antibodies on the Pharmacokinetics and Efficacy of COVID-19 mRNA Vaccine (****Comirnaty) *in vivo***

**Supplementary figures:**

| **Naive Group (n=10)** |  | | |  |  |  | |  |  | |  | |  | |  | |  |  |
| --- | --- | --- | --- | --- | --- | --- | --- | --- | --- | --- | --- | --- | --- | --- | --- | --- | --- | --- |
| **Ear punching identification No.** | **13-UN** | **13-L1** | **13-R1** | | **13-L2** | **13-LR** | **14-UN** | | | **14-L1** | | **14-R1** | | **14-L2** | | **14-LR** | | **Average concentration**  **(μg/mL)** |
| **Anti-PEG IgG concentration (μg/mL)** | **0.00** | **0.00** | **0.00** | | **0.00** | **0.00** | **0.00** | | | **0.00** | | **0.00** | | **0.00** | | **0.00** | | **0.00** |
| **Anti-PEG IgM concentration (μg/mL)** | **0.00** | **0.00** | **0.00** | | **0.00** | **0.00** | **0.00** | | | **0.00** | | **0.00** | | **0.00** | | **0.00** | | **0.00** |
| **Anti-PEG IgG + IgM concentration (μg/mL)** | **0.00** | **0.00** | **0.00** | | **0.00** | **0.00** | **0.00** | | | **0.00** | | **0.00** | | **0.00** | | **0.00** | | **0.00** |
| **Group 1 (n=10)** |  |  |  | |  |  |  | | |  | |  | |  | |  | |  |
| **Ear punching identification No.** | **2-L1** | **8-UN** | **7-R1** | | **12-R1** | **5-UN** | **4-L1** | | | **3-R1** | | **11-UN** | | **5-R1** | | **9-R1** | | **Average concentration**  **(μg/mL)** |
| **Anti-PEG IgG concentration (μg/mL)** | **1.13** | **3.04** | **7.02** | | **0.46** | **0.78** | **13.14** | | | **21.47** | | **12.03** | | **18.88** | | **27.22** | | **10.52** |
| **Anti-PEG IgM concentration (μg/mL)** | **0.17** | **0.23** | **0.27** | | **0.30** | **0.39** | **0.64** | | | **0.18** | | **0.15** | | **0.40** | | **0.19** | | **0.29** |
| **Anti-PEG IgG + IgM concentration (μg/mL)** | **2.30** | **3.27** | **7.29** | | **0.76** | **1.17** | **13.78** | | | **21.65** | | **12.18** | | **19.28** | | **27.41** | | **10.81** |
| **Group 2 (n=10)** |  |  |  | |  |  |  | | |  | |  | |  | |  | |  |
| **Ear punching identification No.** | **4-UN** | **10-R1** | **10-L1** | | **10-UN** | **11-L1** | **9-UN** | | | **12-L1** | | **6-L1** | | **11-R1** | | **2-R1** | | **Average concentration**  **(μg/mL)** |
| **Anti-PEG IgG concentration (μg/mL)** | **45.77** | **41.46** | **32.64** | | **31.51** | **54.60** | **31.28** | | | **26.16** | | **46.75** | | **27.13** | | **29.67** | | **36.70** |
| **Anti-PEG IgM concentration (μg/mL)** | **0.19** | **0.10** | **0.12** | | **0.21** | **0.31** | **0.30** | | | **8.36** | | **52.77** | | **9.74** | | **3.18** | | **7.53** |
| **Anti-PEG IgG + IgM concentration (μg/mL)** | **45.96** | **41.56** | **32.76** | | **31.72** | **54.91** | **31.58** | | | **34.52** | | **99.52** | | **36.87** | | **32.85** | | **44.23** |

**Table 1. The table displayed the concentrations of mouse anti-PEG IgG and IgM**

The concentrations of anti-PEG IgG, IgM and IgG + IgM in plasma samples from mice were determined by comparing them to standard curves of IgG-6.3 and IgM-AGP_4_, respectively. Every mouse’s ear was marked for identification purposes. For instance, the uncut ear in cage 13 would be labeled as “13-UN”, while the left ear that was cut would be labeled as “13-L1”. Naïve group (n = 10), Group 1 (n = 10), Group 2 (n = 10).

**
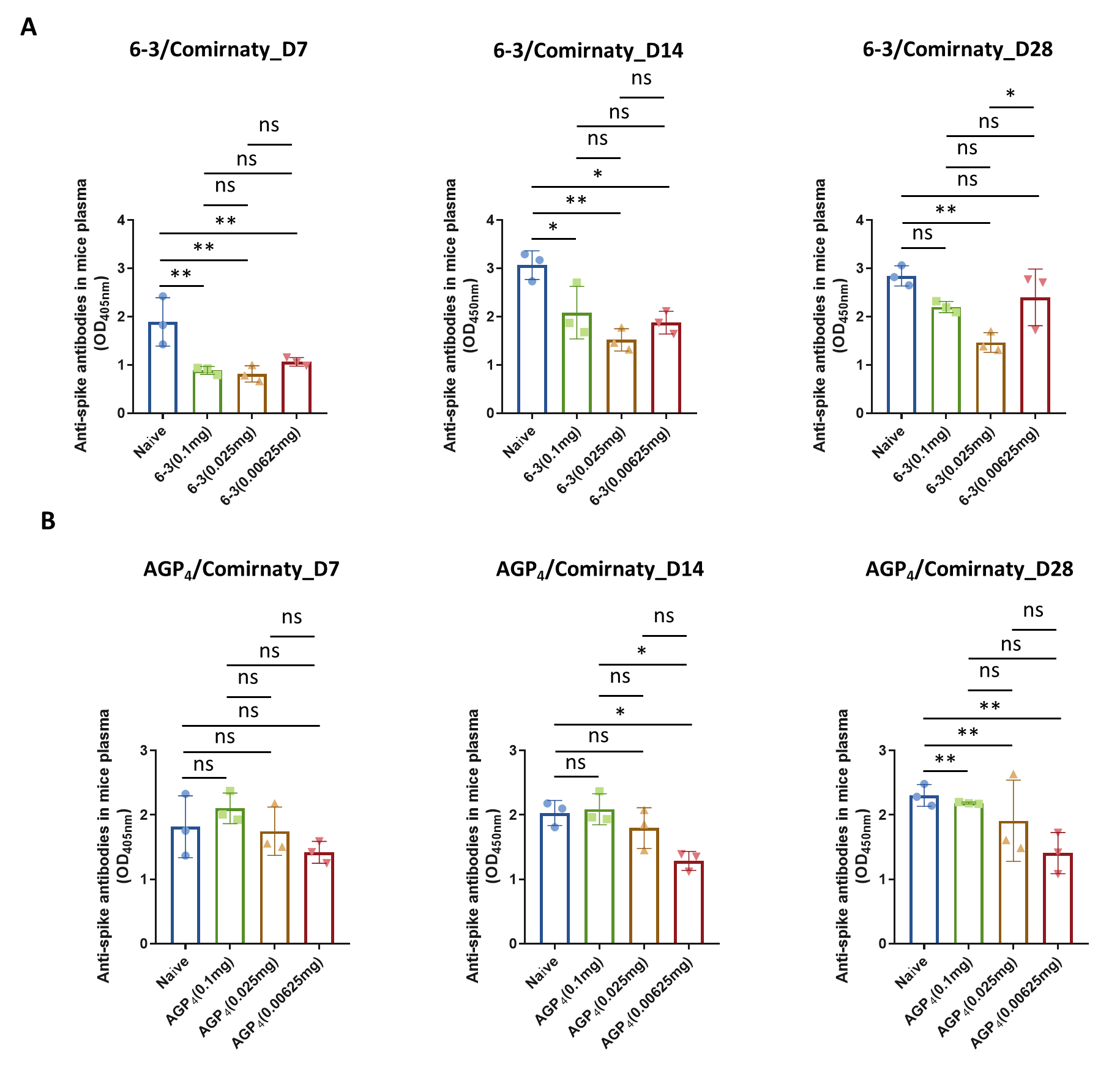
****Supplementary Figure 1. Influence of anti-PEG antibodies on the efficacy of 1^st^ dose mRNA vaccine in mice**

We intravenously injected different concentrations of anti-PEG antibodies to establish a passively transferred anti-PEG antibody mouse model. **(A)** Comparison of the absorbance OD_405nm_ of anti-spike antibodies between injected anti-PEG antibody (mouse anti-PEG IgG1, clone 6.3, total injected antibody amount ■: 0.1 mg, ▲:0.025 mg, ▼: 0.00625 mg) groups and the PBS group (●) after Comirnaty injection 7, 14, and 28days. **(B)** Comparison of the absorbance OD_405nm_ of anti-spike antibodies between the injected anti-PEG antibody (mouse anti-PEG IgM, clone AGP_4_, total injected antibodies amount was ■: 0.1 mg, ▲:0.025 mg, ▼: 0.00625 mg) groups and the PBS group (●) after Comirnaty injection 7, 14, and 28days. (n = 3) Statistical analysis was performed by ordinary one-way ANOVA. *, p < 0.05, **, p < 0.01, ***, p < 0.001 as
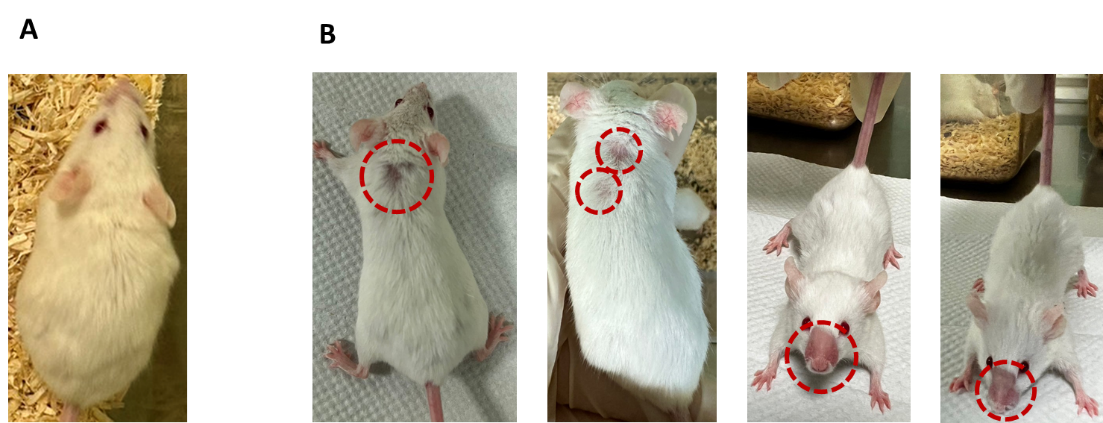
compared to the naïve group.

**Supplementary Figure 2. We observed an Alopecia Areata-like phenomenon in the endogenous anti-PEG antibody mouse model.**

**(A)** Normal mouse. **(B)** Mice who exhibited hair loss (red circles).
